# An assessment of mosquito collection techniques for xenomonitoring of anopheline-transmitted Lymphatic Filariasis in Ghana

**DOI:** 10.1017/S0031182018000938

**Published:** 2018-06-14

**Authors:** Millicent Opoku, Corrado Minetti, Worlasi D. Kartey-Attipoe, Sampson Otoo, Joseph Otchere, Bruno Gomes, Dziedzom K. de Souza, Lisa J. Reimer

**Affiliations:** 1 Vector Biology Department, Liverpool School of Tropical Medicine, Pembroke Place, L3 5QA, Liverpool, UK; 2 Department of Parasitology, Noguchi Memorial Institute for Medical Research, University of Ghana, Legon, Ghana

**Keywords:** *Anopheles* gravid trap, lymphatic filariasis, microfilaria, xenomonotoring

## Abstract

Monitoring vectors is relevant to ascertain transmission of lymphatic filariasis (LF). This may require the best sampling method that can capture high numbers of specific species to give indication of transmission. Gravid anophelines are good indicators for assessing transmission due to close contact with humans through blood meals. This study compared the efficiency of an Anopheles gravid trap (AGT) with other mosquito collection methods including the box and the Centres for Disease Control and Prevention gravid, light, exit and BioGent-sentinel traps, indoor resting collection (IRC) and pyrethrum spray catches across two endemic regions of Ghana. The AGT showed high trapping efficiency by collecting the highest mean number of anophelines per night in the Western (4.6) and Northern (7.3) regions compared with the outdoor collection methods. Additionally, IRC was similarly efficient in the Northern region (8.9) where vectors exhibit a high degree of endophily. AGT also showed good trapping potential for collecting *Anopheles melas* which is usually difficult to catch with existing methods. Screening of mosquitoes for infection showed a 0.80–3.01% *Wuchereria bancrofti* and 2.15–3.27% *Plasmodium* spp. in *Anopheles gambiae*. The AGT has shown to be appropriate for surveying *Anopheles* populations and can be useful for xenomonitoring for both LF and malaria.

## Introduction

Lymphatic filariasis (LF) is a neglected tropical disease that causes debilitating, acute and chronic morbidities in affected individuals. It is caused by three mosquito-borne parasitic worms: *Wuchereria bancrofti*, which accounts for 90% of cases recorded globally (Ottesen, 2006), *Brugia malayi* and *Brugia timori* accounting for the remaining 10%. LF is present in over 80 countries in the Americas, Asia, the Pacific and Africa (Molyneux *et al*., 2003). It is estimated that 120 million of the world's population is infected, while 40 million suffer from disabilities and psychological trauma due to stigmatization (Brady, 2014; Ichimori *et al.*, 2014; WHO, 2015). It is transmitted by mosquitoes belonging to the *Aedes*, *Anopheles*, *Culex, Mansonia* and *Ochlerotatus* genera (de Souza *et al.*, 2012). In Africa, where *W. bancrofti* is the parasite responsible for LF, anophelines (*Anopheles gambiae s.l.* complex and *Anopheles funestus*) are the main vectors in rural areas across the continent while culicines (*Culex quinquefasciatus*) are the primary vectors in urban areas in eastern and southern parts of Africa (Pederson, 2008). More recently, *Mansonia* mosquitoes have been incriminated as potential vectors in rural Africa as well (Ughasi *et al.*, 2012).

The Global Programme to Eliminate Lymphatic Filariasis (GPELF) was launched in 2000, with the primary goal to interrupt LF transmission through annual mass drug administration (MDA) with albendazole in combination with either ivermectin in Africa or diethylcarbamazine in areas outside Africa (Ottesen, 2006). The GPELF has achieved great successes since its inception, with the elimination of LF in Cambodia, Cook Islands, Maldives, Niue, Sri Lanka, Togo and Vanuatu (Budge, 2014; WHO, 2016, 2017). In addition, 13 countries are now in the post-elimination phase.

According to the WHO guidelines, transmission assessment surveys (TAS) support the decision to stop MDA (WHO, 2011) based on microfilariae (mf) prevalence <1% or antigen prevalence <2% (Stolk *et al.*, 2003). The tools available for transmission assessment include; immunochromatograhic test (ICT) [such as filarial test strip (FTS)], ELISA, polymerase chain reaction (PCR) and mf detection by microscopy (WHO, 2011). However, these tools require blood collection from large numbers of community volunteers. Further, the cross-reactivity of *Loa loa* and *W. bancrofti* reported by Wanji *et al*. (2015) in Cameroon, questions the reliability of ICT especially in parts of Central Africa where these parasites are co-endemic. Meanwhile, monitoring vectors for the presence of parasite DNA (xenomonitoring) is an important assessment tool for LF elimination programmes, with the advantage that it provides a real-time estimate of microfilaria in the community members (Okorie and de Souza, 2016). Mosquito surveillance studies have shown to be useful in assessing the transmission of LF in the Pacific (Chanteau *et al.*, 1994; Burkot and Ichimori, 2002; Farid *et al.*, 2007). Furthermore, studies in Africa show that xenomonitoring can provide valuable information to the LF elimination programme (Chanteau *et al.*, 1994; Boakye *et al*., 2004; Ughasi *et al.*, 2012; de Souza *et al.*, 2014). The traditional methods for xenomonitoring include dissecting vectors for the different developmental stages of the worm. However, as microfilariae (mf) prevalence in human populations becomes low due to MDA, the time and cost involved in processing such large numbers remain a challenge (Burkot and Ichimori, 2002). In this instance, molecular xenomonitoring, a method which allows for processing samples within a shorter time with high precision will improve sample processing (Derua *et al.*, 2017). Additionally, targeting epidemiologically relevant mosquitoes, i.e. older mosquitoes (blood fed, gravid or parous mosquitoes) will further enhance the chances of detecting parasite DNA (Springer *et al.*, 2016). Gravid traps have shown to be useful for such purposes (Mboera *et al.*, 2000). The Centres for Disease Control and Prevention (CDC) gravid traps are routinely used for surveying culicine populations (Williams and Gingrich, 2007; Facchinelli *et al.*, 2008), however, the attractive ovoposition substrate is not effective in collecting anophelines. Recent work by Lindh *et al*. (2016) point to the potential of the *Anopheles* gravid trap (AGT) (OviART) in collecting gravid *Anopheles* mosquitoes. Other mosquito collection tools for monitoring mosquito populations include indoor and outdoor collection methods (e.g. the human landing catches, pyrethrum spray catches (PSC), aspiration of resting mosquitoes, exit traps (ET), barrier nets, box gravid trap (BOX), CDC-light trap (LIT), and the BioGents-sentinel trap). The traps exploit different mosquito behaviours, such as feeding and resting, and habitats with varying sensitivities.

The aim of our study was to assess the efficiency of the AGT for xenomonitoring purposes in two LF endemic areas in Ghana – the coastal Western region and the Northern region. The study evaluated the mosquito composition, density and physiological state of mosquitoes collected from this trap compared with five other collection methods, as well as *W. bancrofti* and *Plasmodium* spp. DNA positivity in the mosquitoes collected.

## Materials and methods

### Study sites

**Fig. 1.**
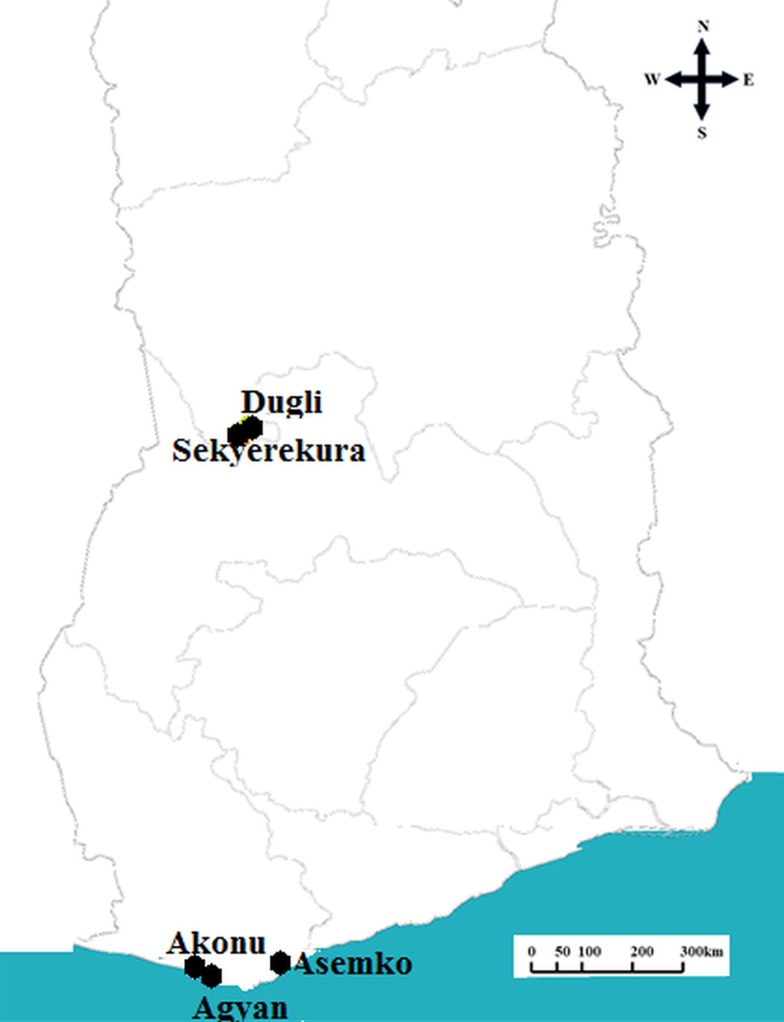
Map of Ghana showing the locations of study villages sampled.

Our study was conducted in villages across the Western Region (Akonu, Agyan and Asemko) and Northern Region (Dugli and Sekyerekura) of Ghana (Fig. 1). Akonu and Agyan are neighbouring communities located in the Nzema East district while Asemko is in the Ahanta West district. Dugli and Sekyerekura are neighbouring communities in the Bamboi district. The major vectors of LF in the Northern savanna region are *An. gambiae*, *Anopheles arabiensis* and *Anopheles funestus* while in the coastal areas, the predominant vectors are *A. gambiae s.s*, *Anopheles melas* and *An. funestus* (de Souza *et al*., 2010). The Northern region is characterized by a rainy season which occurs between May and September and a dry season between December and April with temperatures as high as 40 °C. The Western region has a wet season that spans from April to November and a dry season from December to March.

### Epidemiological survey

An epidemiological survey was conducted to determine the LF prevalence in Agyan (May 2016), Akonu and Asemko (January 2017), Dugli and Sekyerekura (October 2017). Finger-prick blood was collected from volunteers aged 16 years and above and tested using the FTS (Alere™) to detect circulating filarial antigen. Positive individuals were then followed up for the presence of microfilariae through the collection of blood between 10 pm and 2 am. This coincides with the peak time of mf in peripheral blood. Blood samples were screened by microscopy using the counting chamber technique described by Agbolade and Akinboye (2005).

### Mosquito collection methods

The AGT was designed and described by Dugassa *et al.* (2013). It was made of a rectangular basin measuring 45 cm × 33 cm × 11.5 cm (length × width × height), with a 4 cm hole on the side and 6 L rectangular basin. An open plastic tube (collection chamber) was inserted into the hole and the other opening of the tube was sealed with fibre glass netting to prevent trapped mosquitoes from escaping. The tube was placed and secured halfway into an aluminium collapsible pipe. The flexible tube was connected to a 12 V fan that provided suction on the water surface. The efficiency of AGT was compared with two gravid traps; BOX-the Box gravid trap (by BioQuip, Rancho Dominguez, CA) and CDC-gravid trap (Model 1712 from John W. Hock Company), and LIT (by UPL limited) for outdoor collections. The AGT was also evaluated against the BGS-BioGents-sentinel, ET-Exit traps, IRC-Indoor resting collection and the PSC-pyrethrum spray catches which were used to collect mosquitoes indoor. The BGS (BioGents) was baited with the Anopheles odour lure. The LIT was baited with cotton wool soaked in 1-Octen-3-ol. The IRC were done using a battery-powered aspirator (Vazquez-Prokopec *et al.*, 2009), with the interior ceiling, walls and any hanging clothing aspirated. The AGT, BOX, CDC, ET, LIT and BGS were set up at 6 pm and removed the following morning at 6 am. PSCs were conducted between 5:30 am and 6 am in selected houses in the Western region. Whereas, the IRCs were conducted from 5:30 am to 8:30 am in all households in the villages in the Northern region. Water from larval habitats was used in all the gravid traps. Only valid collection nights (nights where all the traps worked through the night) were used in comparisons. The data collected on days where batteries of one or more of the traps failed were excluded.

### Western region mosquito collection

The AGT was compared with CDC, BOX, LIT, ET and PSC in the three Western region villages. Mosquito collections were conducted in March and May 2017 with a total of 26 collection nights each for AGT, BOX, CDC and LIT; 18 for ET and 37 for PSC in 13 consecutive nights. The four outdoor collection methods (AGT, CDC, BOX and LIT) were rotated among selected locations within the densely populated sections of the villages and larval habitats. The two indoor methods (ET and PSC) were done in randomly selected rooms with at least one sleeper.

### Northern region mosquito collection

The AGT was compared with the BOX, BGS and IRC in the Northern region. Mosquito collections were conducted in October 2017 with 12 collection nights for each trap type. The three outdoor traps (AGT, BOX, BGS) were rotated among selected locations within each village. Each location consisted of a family compound and IRCs were conducted in all rooms which were occupied by a sleeper the night before (the number of rooms per compound ranged 1–8). The mean number of mosquitoes per room is presented for each location (Fig. 5).

### Mosquito processing

The collected mosquitoes were identified morphologically using keys by Gillies and Coetzee (1987). Mosquitoes collected from the Western region were scored based on abdominal status – fed, unfed or gravid. A proportion of the mosquitoes collected from the Western region were dissected and ovaries removed and dried to determine parity based on ovary tracheation as described by Detinova (1962). Mosquito legs were used for molecular identification of members of the *An. gambiae s.l.* complex, based on restriction fragment length polymorphism described by Fanello *et al.* (2002). Mosquitoes from the Northern region were only identified using morphological features.

### Wuchereria bancrofti *and* Plasmodium *detection in mosquitoes*

The anopheline mosquitoes that were in good condition (not damaged) were screened for *W. bancrofti* and *Plasmodium* spp. DNA, in pools of up to 5 mosquitoes based on trap type, species and location. The heads and thoraces were screened separately from the abdomens. Genomic DNA (gDNA) was extracted from the mosquito pools, using the Livak extraction method (Livak, 1984). To determine the presence of *W. bancrofti* DNA, the ITS1 gene in the 18S and 5.8S subunits of the rRNA from filarial worms was amplified as described by Jiménez *et al.* (2011). For the identification of *Plasmodium* spp. DNA, the COX-1 gene was amplified in a single step PCR as described by Echeverry *et al.* (2017).

### Data analysis

Morphological and molecular identification showed no differences in species composition between villages of the same region, therefore, data were combined for statistical analysis using SPSS (IBM24). To evaluate the trapping efficiency of AGT, a Bonferroni posthoc analysis of variance (ANOVA) was performed to compare the mean number of *Anopheles* mosquitoes caught by each method, per night.

Trapping efficiency was also evaluated by comparing an estimated mean number of *Anopheles* that were likely to have taken a blood meal in their lifetime since these are the target population for xenomonitoring. The proportion of mosquitoes that were unfed but parous, blood fed and gravid mosquitoes collected in each trap was estimated and multiplied by the mean catch per collection/night to get the estimated number that had previously taken a blood meal.

The prevalence of *W. bancrofti* and *Plasmodium* spp. DNA in mosquitoes was estimated using the PoolScreen software 2.0 (Katholi *et al.*, 1995) and the maximum likelihood estimates reported with 95% confidence interval (CI).

## Results

### LF infection prevalence

Filarial antigen prevalence ranged 12.2–27.9% in the study villages and microfilaria prevalence ranged 1.5–3.8% (Table 1).

**Table 1.** Estimated CFA and mf prevalence in the five villages

Region	Community	Number tested for CFA	CFA prevalence (%)	Estimated village microfilariae prevalence (%)
Western	Agyan	195	23.0	2.2
Western	Akonu	93	27.9	1.5
Western	Asemko	115	12.2	1.7
Northern	Dugli	78	19.2	2.6
Northern	Sekyerekura	79	15.2	3.8

### Mosquito composition

#### Western region

**Fig. 2.**
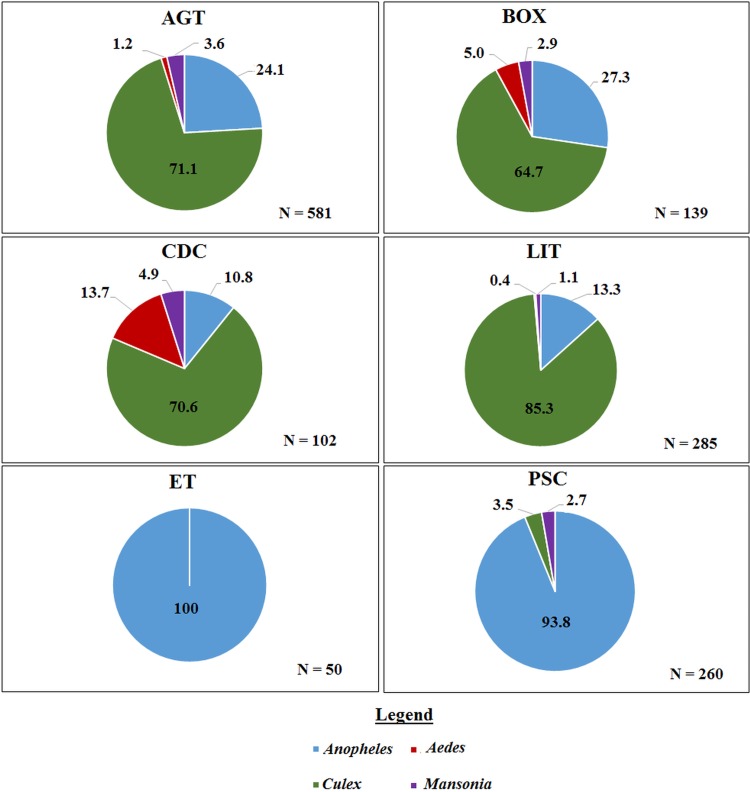
The proportion of mosquito genera caught per trap type in the Western region. Where ‘*N*’ is the number of mosquitoes caught in each trap. AGT, Anopheles gravid trap; BOX, Box gravid trap; CDC, CDC gravid trap; ET, Exit trap; LIT, Light trap; PSC, Pyrethrum spray catch.

A total of 1417 mosquitoes was collected in the Western region. Morphological identification of collected mosquitoes showed that 4.6% were *Aedes* spp, 36.8% were *A. gambiae s.l.*, 58.4% were *Culex* spp and 0.2% were *Mansonia* spp. There was no significant difference in the species composition across the three villages. The largest proportion of mosquitoes caught using the outdoor collection methods (AGT, BOX, CDC and LIT) were *Culex* whereas, the largest proportion of the total catch in ET and PSC were *Anopheles* (Fig. 2). Molecular characterization of the 442 *An. gambiae s.l.* collected from the villages in the Western region, show that 44.0% were *An. gambiae s.s.*, 43.8% were *An. melas*, while 12.1% did not amplify. Whereas in the Northern region, 55.1% of 78 *Anopheles* were identified as *An. gambiae s.s*. however, 44.9% did not amplify with the *An. gambiae* complex primers used (primers included; *An. arabiensis, An. gambiae* and *An. melas*). These proportions did not differ between the indoor and outdoor collection methods (*χ*^2^ = 0.16, *P* = 0.7).

#### Northern region

**Fig. 3.**
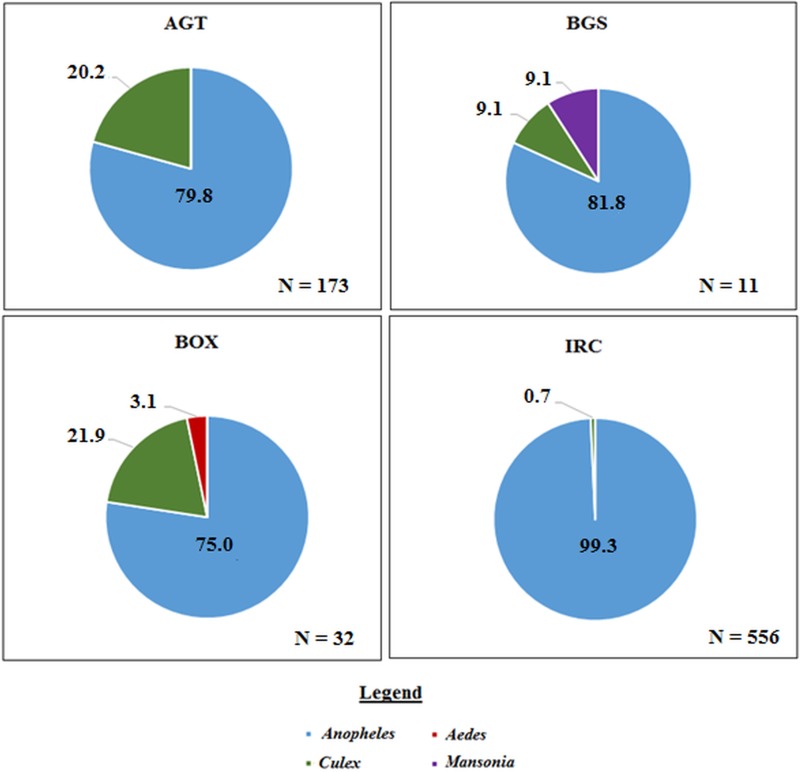
The proportion of mosquito genera caught per collection method in the Northern region. Where ‘*N*’ is the number of mosquitoes caught in each trap. AGT, Anopheles gravid trap; BGS, BG sentinel trap; BOX, Box gravid trap; IRC, indoor resting collection.

A total of 771 mosquitoes was collected in the Northern region. Of the 555 mosquitoes collected indoors, 473 (85.2%) were *An. gambiae* sl, and 68 (12.2%) were *An. funestus*, 10 (1.8%) were other *Anopheles* spp. including *Anopheles rufipes* and *Anopheles coustani* and 4 (0.7%) were *Culex* spp (Fig. 3).

### Comparison of the trapping efficiency by mosquito composition and density

**Fig. 4.**
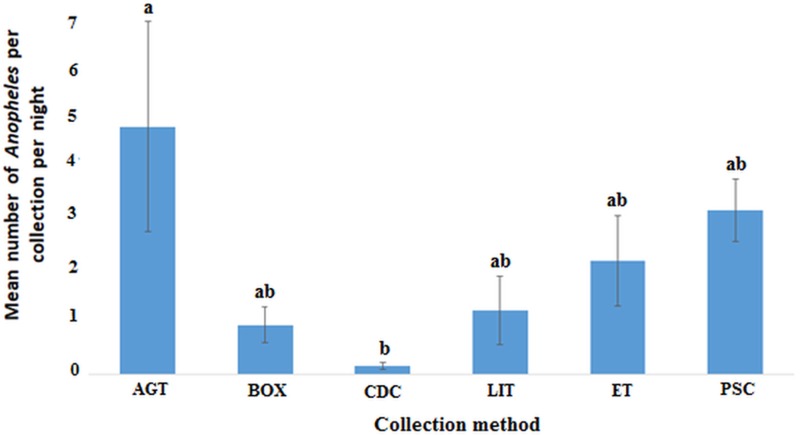
Plots of the mean number of anopheline mosquitoes caught by each method per night in the Western region (*P* < 0.05). Bars with identical letters are not significantly different from each other. Error bars show standard error of the mean.

In the Western region, AGT caught the highest mean number of anophelines per collection night (4.62, 95% CI 0.49–8.74) followed by PSC (3.05, 95% CI 1.85–4.26) and the least being CDC (0.15, 95% CI 0.01–0.30). Pairwise comparisons show that the differences in the mean catch per collection night of anophelines was only significant for AGT when compared with CDC, *F*_(5, 153)_ = 2.959, *P* = 0.014 (Fig. 4).

**Fig. 5.**
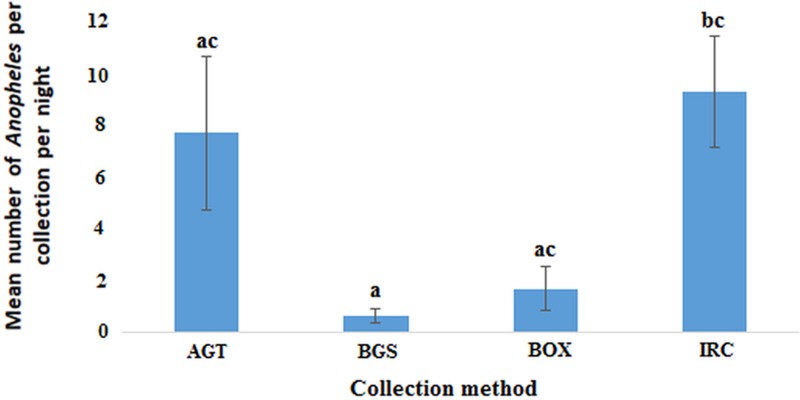
Plots of the mean number of anophelines caught by each method per night in the Northern region (*P* < 0.05). Bars with identical letters are not significantly different from each other. Error bars show standard error of the mean.

In the Northern region, the indoor resting collections had the highest mean number of anophelines per collection night (8.86, 95% CI 4.13–13.59) followed by AGT (7.33, 95% CI 0.83−13.831) with BGS catching the least (0.58, 95% CI 0.01–1.16). The observed differences in the mean number of anophelines was statistically significant between IRC and BGS but not AGT and BOX (*F*_(3, 44)_ = 4.808; *P* < 0.01) (Fig. 5). However, the differences in mean between AGT and BGS, AGT and BOX or BGS and BOX were not statistically significant.

### Trapping efficiency for mosquitoes that have previously taken a blood meal

Estimating the number of mosquitoes that were likely to have previously taken a blood meal is important for xenomonitoring. These mosquitoes include those that were caught bloodfed, gravid or unfed and parous. The parity dissections were only conducted in the Western region study. The results show that AGT caught the highest mean number of *An. gambiae s.l.* previously exposed to at least one blood meal. PSC was second with CDC collecting the least number per collection night. As expected, the gravid traps caught a high proportion of gravid *An. gambiae s.l.* while the indoor collections caught a high proportion on bloodfed *An. gambiae s.l.* (Table 2).

**Table 2.** Estimated mean number of *A. gambiae s.l.* from the Western region which have previously taken a blood meal

Collection method	Mean catch^a^ (±s.e.)	Proportion unfed nulliparous	Proportion unfed parous	Proportion bloodfed	Proportion gravid	Estimated mean number exposed to blood meal
AGT	4.60 (1.96)	0.12	–	0.311	0.57	4.05
BOX	0.92 (0.33)	0.13	0.13	0.105	0.63	0.79
CDC	0.15 (0.07)	–	–	0.5	0.5	0.15
LIT	1.19 (0.64)	0.64	0.3	0.03	0.03	0.43
ET	2.11 (0.84)	0.13	0.17	0.46	0.24	1.84
PSC	3.05 (0.59)	0.16	0.046	0.66	0.13	2.55

AGT, Anopheles gravid trap; BOX, Box gravid trap; CDC, CDC-gravid trap; LIT, light trap; ET, Exit trap and PSC, Pyrethrum spray catches.

a Estimates per collection night in the case of AGT, BOX, CDC, LIT, ET and per room for PSC.

### Infection prevalence in mosquitoes

Pools of heads and thoraces were split from abdomens and screened separately for *W. bancrofti* and *Plasmodium* spp DNA. The results are presented in Table 3. In the Western region, none of the head/thorax pools were positive for either. However, *W. bancrofti* and *Plasmodium* spp. DNA were detected in pools of mosquito abdomens. A 0.80% and 2.15% prevalence of *W. bancrofti* and *Plasmodium* spp. was observed in the *An. gambiae* collected from the Western region. These included mosquitoes (i.e. 3 pools of 15 mosquitoes) that had been captured using PSC (the only method that had mosquitoes that were positive for *W. bancrofti*). The *Plasmodium* positives pools were obtained from 3, 4 and 1 pool of 14, 11 and 2 mosquitoes that were caught in AGT, BOX and ET, respectively. The *Plasmodium* spp. positives were recovered from the pools that contained unfed, bloodfed and gravid *Anopheles* mosquitoes, indicating that, immature stages of the parasite were present in the midgut. Unsurprisingly, the *W. bancrofti* DNA positive pools were all from those that were bloodfed. Screening of pools of *Mansonia* show a prevalence of 3.57% (95% CI 0.11–17.08%) *Plasmodium* spp. and 3.86% (95% CI 0.12–18.45) of *W. bancrofti* DNA. In the Northern region, we observed a higher overall prevalence of *W. bancrofti* at 3.01% (95% CI 1.60–5.05) and *Plasmodium* spp. at 3.27% (1.78–5.40). The *W. bancrofti* positive pools were from 2, 3 and 7 pools of 5, 9 and 29 for AGT, BOX and IRC, respectively. While that of *Plasmodium* spp. were from 1 to 11 pools of 5 and 52 mosquitoes that were captured using BOX and IRC, respectively. In addition, a small number of head/thorax pools were positive for *W. bancrofti* (0.3%, 95% CI 0.01–1.5) and *Plasmodium* spp. (0.9%, 95% CI 0.2–2.5), indicating the developing stage of filarial worms were present in the thorax, and the infective stage sporozoites were present in the salivary glands. The positive mosquitoes were collected from IRC and AGT and these mosquitoes were *An. gambiae* and *Culex* spp.

**Table 3.** Infection prevalence of *W. bancrofti* and *Plasmodium* spp. in mosquito pools

Region	Total number of mosquitoes screened	Estimated prevalence *W. bancrofti* DNA (%) (95% CI)	Estimated prevalence Plasmodium spp. DNA (%) (95% CI)	Trap type
Western	382	0.80 (0.155–2.30)	2.15 (0.867–4.28)	PSC (Wb) AGT, BOX, ET (Plas)
Northern	516	3.01 (1.60–5.05)	3.27 (1.78–5.40)	IRC, BOX, AGT (Wb) IRC, BOX (Plas)

a The table above shows the estimated prevalence of *W. bancrofti* and *Plasmodium* spp. DNA in the mosquitoes collected using PoolScreen 2.0.

## Discussion

Monitoring parasite infection in vector populations can be used to assess transmission as well as infection in the human population (Boakye *et al*., 2004; Ughasi *et al*., 2012; de Souza *et al.*, 2014). However, there is a challenge identifying efficient vector collection methods for anopheline mosquitoes, the primary vectors in sub-Saharan Africa for both LF and malaria. Using gravid traps increases the chance of catching infected mosquitoes as gravid mosquitoes would have taken at least one blood meal (Irish *et al.*, 2013). CDC gravid traps, purposely designed for *Culex* mosquitoes, are used routinely for the surveillance of diseases such as dengue fever and lymphatic filariasis transmitted by *Culex* mosquitoes (L'Ambert *et al.*, 2012; Hapairai *et al.*, 2015). However, an equivalent for anophelines is not widely used for monitoring, and this has been a challenge for surveying these populations. This study reports on the first evaluation of an *Anopheles* gravid trap for monitoring LF and malaria in vectors in Ghana.

In the Western region, about 40% of the total *An. gambiae s.l.* collected were *An. melas*. *Anopheles melas* is usually associated with mangroves, which were present in the study communities. Tuno *et al*. (2010) also reported on the abundance of *An. melas* in the western coast of Ghana. In the Northern Region, the *Anopheles* sampled included *An. coustani*, *An. funestus*, *An. rufipes* and *An. gambiae s.l.* which are also implicated in malaria transmission (Tabue *et al.*, 2017). These *Anopheles* species have been reported to exhibit ‘limitation’ which favours transmission even when mf prevalence is low (McGreevy *et al.*, 1978; Bryan, 1990; Amuzu *et al.*, 2010).

For this study, trapping efficiency was evaluated by comparing the mean number of *Anopheles* per trap night. Amongst the gravid traps used, the AGT performed better than the Box gravid trap (BOX) and CDC-gravid trap (CDC), even though all three were baited with water from larval habitats. AGT had the highest proportion of *An. gambiae s.l* exposed to a blood meal as well as the highest mean number of *An. gambiae s.l* compared with the other collection methods in the Western region. Its efficiency at trapping exposed mosquitoes was approximately 1.6 and 2.2 times better than PSC and ET, respectively. Amongst the outdoor collection methods, it was 5.1, 27 and 9.4 times better than BOX, CDC and LIT. The improved performance of AGT could be due to a bigger fan, which provided a suction effect over the entire water surface. Whereas, the suction effect for the BOX and CDC were only strong at the opening to the collection chambers but the effect was less towards the periphery. AGT resulted in a higher catch than Exit trap (ET), CDC-LIT and pyrethrum spray collection (PSC), when standardized per location per night. Similar observations were made in the testing of AGT in Kenya (Lindh *et al.*, 2016). The Kenyan study evaluated the trapping efficiency of the OviART gravid trap designed to collect gravid *Anopheles*. The OviART gravid trap, which is similar to the AGT used in this study, collected 2.3 times the number of *An. gambiae s.l.* compared with BOX. In our study, the AGT also showed good trapping efficiency for *An. melas*, even though this species prefers to breed in high salinity water compared with other anophelines. AGT also caught the highest mean number of *Anopheles* that had previously taken a blood meal. Similarly, Lindh *et al.* (2016) observed a higher proportion (90%) of gravid mosquitoes in the OviART trap. The main difference in the AGT and OviART trap is the oviposition tray. The AGT had a 6 L rectangular basin unlike the OviART which had 8 L circular basin in which water was kept serving as an attractant for gravid mosquito. The rectangular basin of the AGT provides a larger surface area for oviposition, compared with the circular basin of the OviART. However, it is not clear whether the aforementioned variations, affects the performance of these traps.

Some of the limitations to the use of the AGT are due to bulkiness and the use of a 12 V car battery which limits portability. There is no protective shield on the traps and the basins get flooded during rains wetting the collection chambers and trapped mosquitoes. Rains can also damage the fan and the battery. As such, improved designs aimed at protecting the components of the trap while also improving portability will make it more useful in the field.

The high numbers and the different species caught in the AGT show that, not only is it efficient for sampling LF and malaria vectors, it can also be employed in sampling other mosquito vectors including *Culex* which is implicated in LF and arbovirus transmission elsewhere (Mak, 2007; Lutomiah *et al.*, 2011; Jones *et al.*, 2012). Information such as vector abundance and diversity within a locality, can help inform local health authorities on vector distribution and implication in the transmission which can be the basis for deploying control measures (Ciota and Kramer, 2013; Oduola *et al*., 2013). Further, parasite positivity in mosquitoes collected with these traps indicates the presence of infection in the community, providing information that may require intensified efforts to manage and control vector-borne diseases (Kouassi *et al*., 2015).

None of the head/thorax pools of mosquitoes were positive for *W. bancrofti* and *Plasmodium* spp. in the Western region, suggesting that these mosquitoes were not carrying any infective stages. However, a small number of pools were positive from the Northern region. The mosquitoes that were positive for *W. bancrofti* DNA were *An. coluzzii* and *An. melas*, the primary vectors of *W. bancrofti* in these areas (de Souza *et al.*, 2010). The number of samples analysed was few, however, they illustrate the utility of detecting parasite DNA in mosquitoes, even when infection prevalence is very low in the community. Detection of parasite DNA confirms the presence of infection in humans and indicate ongoing transmission to mosquitoes. Furthermore, the positive pools obtained from the traps, supports the evidence that these methods are useful for sampling epidemiologically relevant mosquitoes (ie. mosquitoes that were exposed to at least a blood meal). Hence, they can be employed in monitoring vector populations which can provide valuable information to support the decision to stop MDA.

However, comparison of the differences in infection prevalence in mosquitoes between collection methods of *Anopheles* species and locations were not performed. This is a limitation as these information are relevant and can inform vector monitoring campaigns.

In conclusion, AGT was a very efficient collection method compared with the other traps in both study regions, but particularly in the Western region where few mosquitoes were found resting indoors. We found that, on average, AGT collected over 2 times as much as blood exposed *Anopheles* compared with the indoor methods and 5–27 times compared with the other gravid traps. The collection of indoor resting mosquitoes, either by PSC or mechanical aspiration is efficient in areas with high numbers of indoor resting anophelines such as in the Northern region since many rooms can be screened for indoor resting mosquitoes by one team of collectors. While the AGT showed efficiency in trapping mosquitoes, there are limitations to the number of traps that can be set and rotated by a team because of the heavy car battery, which needs to be recharged every few days.
